# Metabolomics Analysis of Skeletal Muscles from FKRP-Deficient Mice Indicates Improvement After Gene Replacement Therapy

**DOI:** 10.1038/s41598-019-46431-1

**Published:** 2019-07-11

**Authors:** Charles Harvey Vannoy, Victoria Leroy, Katarzyna Broniowska, Qi Long Lu

**Affiliations:** 1 McColl-Lockwood Laboratory for Muscular Dystrophy Research, Carolinas Medical Center, Atrium Health, Charlotte, NC 28203 USA; 2 Metabolon, Inc., Morrisville, NC 27560 USA

**Keywords:** Predictive markers, Metabolomics

## Abstract

Muscular dystrophy-dystroglycanopathies comprise a heterogeneous and complex group of disorders caused by loss-of-function mutations in a multitude of genes that disrupt the glycobiology of α-dystroglycan, thereby affecting its ability to function as a receptor for extracellular matrix proteins. Of the various genes involved, *FKRP* codes for a protein that plays a critical role in the maturation of a novel glycan found only on α-dystroglycan. Yet despite knowing the genetic cause of *FKRP*-related dystroglycanopathies, the molecular pathogenesis of disease and metabolic response to therapeutic intervention has not been fully elucidated. To address these challenges, we utilized mass spectrometry-based metabolomics to generate comprehensive metabolite profiles of skeletal muscle across diseased, treated, and normal states. Notably, *FKRP*-deficient mice elicit diverse metabolic abnormalities in biomarkers of extracellular matrix remodeling and/or aging, pentoses/pentitols, glycolytic intermediates, and lipid metabolism. More importantly, the restoration of FKRP protein activity following AAV-mediated gene therapy induced a substantial correction of these metabolic impairments. While interconnections of the affected molecular mechanisms remain unclear, our datasets support the notion that global metabolic profiling can be valuable for determining the involvement of previously unsuspected regulatory or pathological pathways as well as identifying potential targets for drug discovery and diagnostics.

## Introduction

Muscular dystrophy-dystroglycanopathies constitute a clinically heterogeneous group of muscular dystrophies that arise from primary (*DAG1* (OMIM 128239), which encodes dystroglycan), secondary (genes participating in the maturation of glycans on α-dystroglycan), or tertiary (genes involved in the fabrication of the carbohydrate building blocks) genetic defects associated with the processing and functionality of α-dystroglycan^1,2^. Mutations in one of the genes involved, *FKRP* (OMIM 606596), cause rare forms of limb-girdle muscular dystrophy (LGMD) with a broad spectrum of clinical manifestations characterized by progressive weakness and degeneration of the skeletal muscles that may include some cardiovascular and/or neurological defects^3–6^. These genetic mutations account for approximately 9% of the LGMD population^7,8^ and result in a complete or partial loss of protein function that affects the incorporation of a ribitol phosphate group into the biosynthetic pathway of a novel *O*-mannosyl glycan (only known to be found on α-dystroglycan)^9–11^. As a consequence, subsequent maturation of this glycan into a functional receptor for extracellular matrix proteins is perturbed, creating muscle membrane instability^12^. Recently, research groups, including our own, have conducted multiple studies aimed at finding therapeutic interventions for these disorders, which include gene replacement therapy^13–17^. Study results suggest that the therapeutic effect is dependent on the dosage administered as well as the time-point of therapeutic intervention, with optimization of both factors capable of achieving near-complete restoration of functional glycosylation on α-dystroglycan^16,17^.

Yet despite knowing the genetic cause and some potential avenues for therapeutic intervention, the molecular pathogenesis of *FKRP*-related dystroglycanopathies and the metabolic response to gene replacement therapy have not been fully elucidated. Moreover, as the current landscape of therapeutic development advances and additional therapies become increasingly available for these types of disorders, the necessity for identifying diagnostic and/or predictive molecular biomarkers becomes critical. One way to address these issues is the use of high-throughput metabolomics, which incorporates some of the most advanced approaches to systematic molecular phenotyping and provides an ideal theranostic platform for the discovery of biomarker patterns and potential therapeutic targets^18^. Specifically, the use of analytical tools such as ultra-high performance liquid chromatography-tandem mass spectrometry (UHPLC-MS/MS) can be employed to generate global metabolite profiles that reveal the entirety of low-molecular-weight molecules (e.g., amino acids, carbohydrates, and fatty acids) that are essential components of multiple mammalian systems and are required for energy generation, the biosynthesis of important macromolecules, and maintaining metabolic homeostasis^19^. This strategy has already been evaluated in skeletal/cardiac tissue and serum from animal models and patients exhibiting similar physiological disorders, including Duchenne, Becker, facioscapulohumeral, and other limb-girdle muscular dystrophies^20–23^. However, we hypothesize that there will be a differential representation, to some degree, in the molecular mechanisms and/or metabolic regulation involved in *FKRP*-related dystroglycanopathies, and as a result, these types of disorders should be thoroughly evaluated as well.

In this study, a comprehensive metabolomics-based approach using UHPLC-MS/MS was performed to identify tissue-specific metabolomic impairments associated with *FKRP*-deficient mice and assess the therapeutic responsiveness to our established systemic adeno-associated virus (AAV)-mediated gene therapy approach. Our results demonstrate the feasibility of the metabolomics-based approach and provide a deeper understanding of *FKRP*-related dystroglycanopathies that can be helpful in identifying biomarkers of disease progression, distinguishing molecular markers and targets for therapeutic intervention, as well as predicting long-term, treatment-related side effects.

## Results

This high-throughput metabolomics study across diseased, treated, and normal states is an extension of our previous assessment of protein/gene expression, histopathology, skeletal muscle function, and cardiorespiratory function performed over a 52-week observation period using the same mouse cohorts^17^. Accordingly, this study utilizes our unique glycosylation-deficient mouse model containing a homozygous missense mutation (c.1343 C > T, p.Pro448Leu) in the *FKRP* gene (FKRP^P448L^), as previously described^24,25^. Correction of the FKRP deficiency was accomplished by a single tail vein injection of an AAV serotype 9 vector expressing a codon-optimized, full-length human FKRP coding sequence under control of a muscle-specific creatine kinase-based promoter (abbreviated as AAV9-hFKRP) at a dose of 5 × 10^13^ vg/kg to FKRP^P448L^ mice (n = 6) in an early stage of disease progression (5 weeks of age). Mice were consistently monitored for a 52-week period and then subsequently euthanized for analysis. Age and sex-matched untreated FKRP^P448L^ (n = 6) littermates and wild-type C57BL/6 (n = 6) mice were used as controls in these metabolic studies. All mice remained healthy in appearance, activity, and body weight throughout the study observation period.

### Metabolomic profile of the skeletal muscle across diseased, treated, and normal states

The degree of pathologic change is quite obvious in the hindlimb skeletal muscles of our FKRP^P448L^ mouse model at later stages of disease progression, which recapitulates the clinical severity observed in patients^24^. Consequently, we conducted metabolomics data analysis on quadriceps muscles derived from each cohort of 52-week-old C57BL/6, untreated FKRP^P448L^, and AAV9-hFKRP-treated FKRP^P448L^ mice. Global metabolic profiling using UHPLC-MS/MS was able to identify and quantitate a total of 524 metabolites with known identity, revealing that 247 metabolites (47% of total) were statistically significant (p ≤ 0.05, q ≤ 0.10) and either upregulated or downregulated in a group comparison of untreated FKRP^P448L^ versus C57BL/6, 187 metabolites (36% of total) for AAV9-hFKRP versus untreated FKRP^P448L^, and 99 metabolites (19% of total) for AAV9-hFKRP versus C57BL/6 (Table 1).

**Table 1:** Global metabolic profiling.

Metabolic Pathway	Metabolites Assayed	^a^FKRP^P448L^ versus C57BL/6	^a^AAV9-hFKRP versus FKRP^P448L^	^a^AAV9-hFKRP versus C57BL/6
Amino Acid	111	49 (32)	42	22
Peptide	11	3 (3)	2	1
Carbohydrate	43	25 (14)	11	6
Energy	13	4 (3)	3	2
Lipid	282	132 (74)	111	54
Nucleotide	32	19 (11)	10	8
Cofactor/Vitamin	20	8 (8)	4	2
Xenobiotic	12	7 (7)	4	4
**Total**	**524**	**247**	**187**	**99**
Downregulated		103	134	61
Upregulated		144	53	38

^a^Statistically significant (p ≤ 0.05, q ≤ 0.10) metabolites detected in global metabolic profiling experiments which are determined from comparison tests of the quadriceps muscles derived from 52-week old C57BL/6, untreated FKRP^P448L^, and AAV9-hFKRP-treated FKRP^P448L^ mice. Values in parentheses refer to the number of metabolites in a given pathway that showed cumulative p ≤ 0.05.

In comparison of the untreated FKRP^P448L^ versus C57BL/6 dataset, the metabolic perturbations mapped to all of the defined metabolic pathways, including amino acid (49/111), peptide (3/11), carbohydrate (25/43), energy (4/13), lipid (132/282), nucleotide (19/32), cofactor/vitamin (8/20), and xenobiotic (7/12) metabolism, and account for a large percentage of each (Table 1 and Fig. S1). Furthermore, the number of the altered skeletal muscle metabolites was almost evenly distributed, with 103 (42%) and 144 (58%) metabolites being significantly down- or upregulated, respectively. A similar trend was observed in the comparison of AAV9-hFKRP versus untreated FKRP^P448L^, but most metabolites were downregulated (72%). In contrast, group comparison of AAV9-hFKRP versus C57BL/6 revealed the normalization of 110 metabolites in the AAV9-hFKRP-treated FKRP^P448L^ mice, with large shifts (≥50%, except for xenobiotics) in the number of biochemicals involved in each defined metabolic pathway (Fig. S1 and Supplementary Dataset). Additionally, 25 metabolites were observed to be significantly under or over-corrected after FKRP gene therapy when compared to the C57BL/6 cohort (Supplementary Dataset). Nevertheless, several metabolic impairments persisted even after FKRP gene therapy, with 61 metabolites remaining unchanged and statistically different compared to the C57BL/6 cohort (Supplementary Dataset).

**Figure 1:**
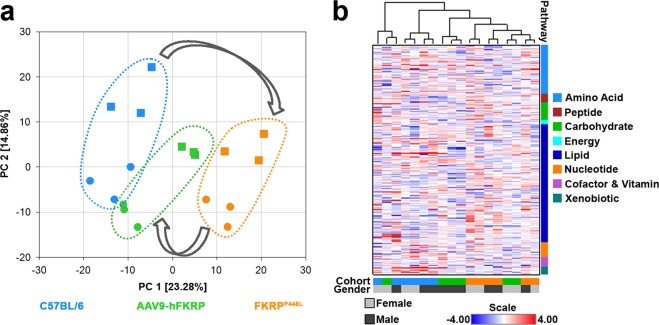
Metabolomic analysis of diseased, treated, and normal skeletal muscle. (**a**) A principal component analysis (PCA) plot and (**b**) a heatmap depicting hierarchical clustering analysis (HCA) from quadriceps muscles derived from 52-week old C57BL/6, AAV9-hFKRP-treated, and untreated FKRP^P448L^ mice were calculated using the Euclidian distance metrics. In the PCA plot, male and female subjects are identified by circles and squares, respectively. In both plots, C57BL/6 samples are identified by blue symbols, AAV9-hFKRP-treated samples by green symbols, and untreated FKRP^P448L^ samples by orange symbols.

Principal component analysis (PCA) effectively and distinctly separated the skeletal muscle samples from each cohort based on phenotype and/or treatment, suggesting that the FKRP deficiency caused a significant change in the overall metabolite profile of the skeletal muscle (Fig. 1a). The PCA revealed good segregation of the C57BL/6 and untreated FKRP^P448L^ skeletal muscle samples on the first principal component (PC 1, x-axis) with the AAV9-hFKRP samples positioned in between these two cohorts, suggesting that FKRP gene therapy can partially reverse the altered metabolism of the dystrophic muscle. More subtle separation along the second principal component (PC 2, y-axis) was observed for each of the skeletal muscle samples and correlates with the gender of the mice. This result is very interesting and suggests that the male mice may respond better to therapeutic intervention and result in a more favorable long-term outcome. Additionally, a hierarchical cluster analysis (HCA) was conducted, generating a heatmap that provides a comprehensive overview of which features are the most distinctive for each cohort (Fig. 1b). In agreement with PCA, HCA also revealed that the skeletal muscle samples from each cohort separated based on phenotype and/or treatment, with all the C57BL/6 samples clustering on the left side of the panel, the untreated FKRP^P448L^ samples clustering on the right side, and AAV9-hFKRP-treated samples being intermixed with the two control cohorts. Once again, this profiling pattern indicates amelioration of the dystrophic phenotype with AAV-mediated FKRP gene therapy. Random Forest classification using named metabolites in C57BL/6 skeletal muscle samples compared to untreated FKRP^P448L^ and AAV9-hFKRP-treated muscle also gave a predictive accuracy of 100% (Table S1). Altogether, these results suggest that FKRP gene therapy can have a significant influence on the metabolic features of the skeletal muscle given that the AAV9-hFKRP cohort is clearly distinguishable from the untreated FKRP^P448L^ cohort. In the following subsections, we will give a comprehensive overview detailing the results of the metabolomics datasets and some of the suspected pathways involved.

### Biomarkers of protein degradation, extracellular matrix remodeling, and/or aging

**Figure 2:**
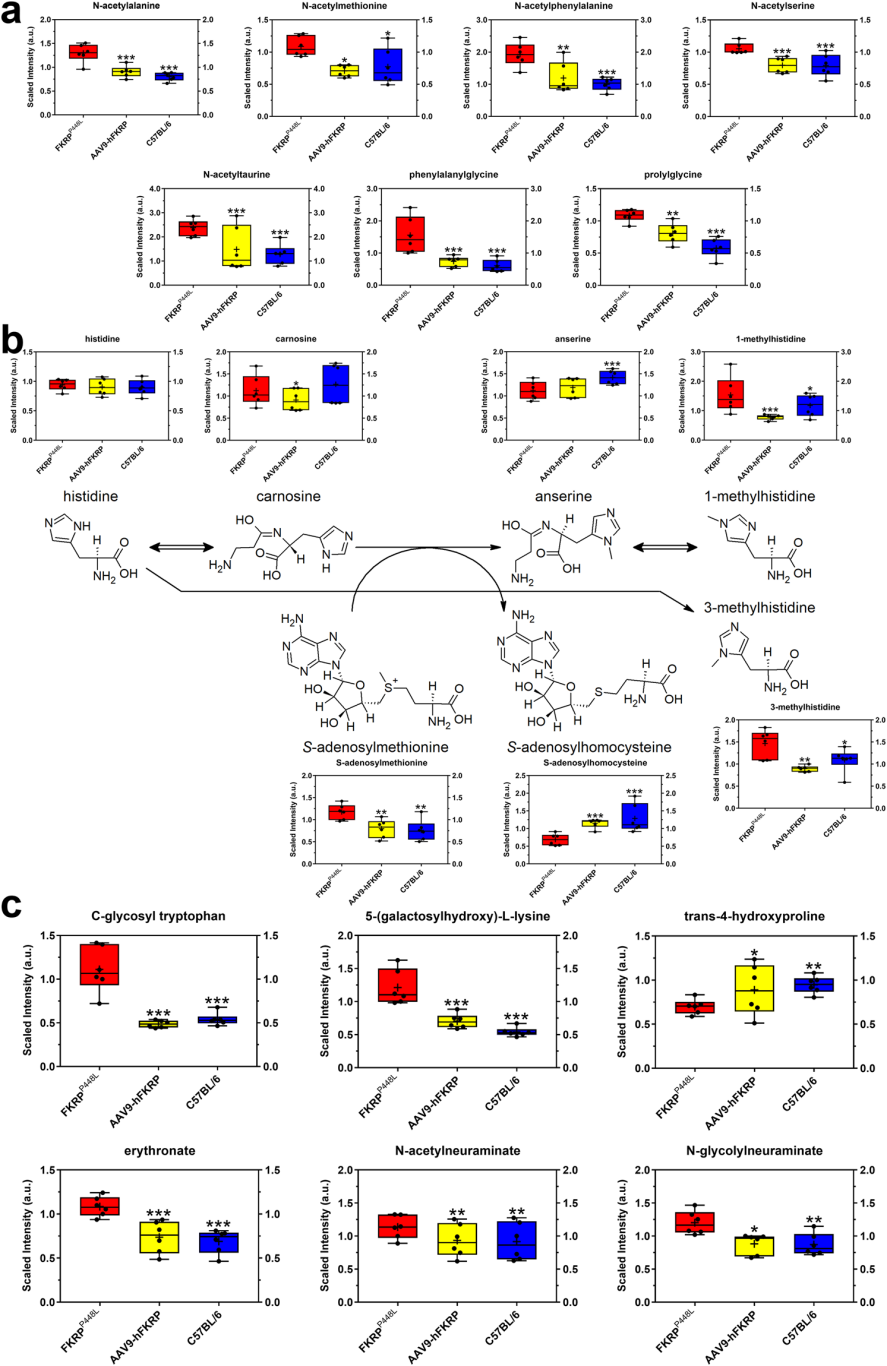
Metabolites associated with protein degradation, extracellular matrix remodeling, and/or aging. Comparison of metabolite abundances associated with (**a**) free acetylated amino acids and dipeptides, (**b**) histidine metabolism, and (**c**) extracellular matrix remodeling and/or aging in quadriceps muscles acquired from 52-week old untreated FKRP^P448L^ (red), AAV9-hFKRP-treated (yellow), and C57BL/6 (blue) mice. *p ≤ 0.05, **p ≤ 0.01, ***p ≤ 0.001; p values were determined by two-way ANOVA with repeated measurements.

Clear physiological differences exist in the FKRP^P448L^ mice, which prompted us to try and identify metabolites associated with protein degradation, extracellular matrix remodeling, and/or aging. Accordingly, several metabolites involved in amino acid metabolism were altered as a result of disease progression. Specifically, levels of numerous acetylated amino acids (e.g., N-acetylalanine (1.61-fold of C57BL/6, p = 2.01 × 10^−5^), N-acetylmethionine (1.41-fold of C57BL/6, p = 2.06 × 10^−2^), N-acetylphenylalanine (1.93-fold of C57BL/6, p = 3.00 × 10^−4^), N-acetylserine (1.33-fold of C57BL/6, p = 3.58 × 10^−5^), and N-acetyltaurine (1.86-fold of C57BL/6, p = 5.00 × 10^−4^)) and dipeptides (e.g., phenylalanylglycine (2.58-fold of C57BL/6, p = 1.28 × 10^−5^) and prolylglycine (1.87-fold of C57BL/6, p = 7.25 × 10^−6^)) were significantly elevated in untreated FKRP^P448L^ skeletal muscles but returned to normal levels after FKRP gene therapy (Fig. 2a). These results could reflect either increased protein breakdown within the skeletal muscle or affected transport of these metabolites into the skeletal muscle from the microenvironment.

Other metabolites that serve as a reliable index of skeletal muscle protein breakdown are those involved in histidine metabolism. Despite unaltered levels of histidine in all cohorts, the levels of methylated histidine derivatives such as 1-methylhistidine (1.30-fold of C57BL/6, p = 2.06 × 10^−2^) and 3-methylhistidine (1.35-fold of C57BL/6, p = 3.66 × 10^−2^) were significantly higher in untreated FKRP^P448L^ skeletal muscles, and FKRP gene therapy was able to reverse this effect (Fig. 2b). Levels of the histidine-derived carnosine (β-alanyl-L-histidine) were also unaffected, but its methylated analog, anserine (β-alanyl-N-methylhistidine), which is typically an abundant dipeptide in skeletal muscle, was significantly reduced in both untreated FKRP^P448L^ (0.79-fold of C57BL/6, p = 9.06 × 10^−6^) and AAV9-hFKRP-treated (0.84-fold of C57BL/6, p = 1.00 × 10^−4^) skeletal muscles.

In *FKRP*-related dystroglycanopathies, the extracellular matrix plays a critical role in development and disease, and as a result, metabolites associated with collagen turnover or remodeling were thoroughly assessed. C-glycosyl tryptophan (2.05-fold of C57BL/6, p = 3.42 × 10^−6^) and 5-(galactosylhydroxy)-L-lysine (2.24-fold of C57BL/6, p = 4.44 × 10^−8^) were significantly elevated and trans-4-hydroxyproline (0.74-fold of C57BL/6, p = 1.20 × 10^−3^) was significantly decreased in untreated FKRP^P448L^ skeletal muscles (Fig. 2c). Because these metabolites are derived from the degradation of proteins bearing post-translationally modified amino acid residues, altered levels of these metabolites may indicate possible changes in collagen homeostasis. Notably, transgenic expression of FKRP corrected the levels of C-glycosyl tryptophan and trans-4-hydroxyproline similar to those observed in the C57BL/6 cohort and significantly decreased 5-(galactosylhydroxy)-L-lysine levels. Additionally, erythronate (1.57-fold of C57BL/6, p = 6.00 × 10^−5^) was also elevated in untreated FKRP^P448L^ skeletal muscles and FKRP gene therapy restored the metabolite levels to normal (Fig. 2c). Increases in erythronate could also indicate augmented extracellular matrix breakdown in *FKRP*-deficient muscle but could also be reporting on the extracellular matrix remodeling process required to improve muscle structure. With regards to muscle aging^26^, levels of sialic acids such as N-acetylneuraminate (1.24-fold of C57BL/6, p = 3.20 × 10^−3^) and N-glycolylneuraminate (1.38-fold of C57BL/6, p = 8.10 × 10^−3^) were significantly elevated in the untreated FKRP^P448L^ skeletal muscles, and FKRP gene therapy was able to restore these levels to normal (Fig. 2c). Furthermore, metabolites associated with purine (e.g., adenosine (1.80-fold of C57BL/6, p = 4.70 × 10^−3^), hypoxanthine (1.50-fold of C57BL/6, p = 2.17 × 10^−2^), inosine 5’-monophosphate (0.87-fold of C57BL/6, p = 5.31 × 10^−6^), and xanthine (1.27-fold of C57BL/6, p = 1.40 × 10^−2^)) and pyrimidine (e.g., orotidine (1.67-fold of C57BL/6, p = 3.35 × 10^−6^), thymidine (0.41-fold of C57BL/6, p = 1.00 × 10^−4^), and uracil (1.46-fold of C57BL/6, p = 1.90 × 10^−3^)) metabolism were significantly altered in the untreated FKRP^P448L^ cohort but were then fully or partially corrected after FKRP gene therapy (except for thymidine), suggesting possible changes in nucleotide catabolism/recycling within the skeletal muscle that may be contributing to cytotoxicity or mutagenicity (Supplementary Dataset).

### Carbohydrate metabolism

**Figure 3:**
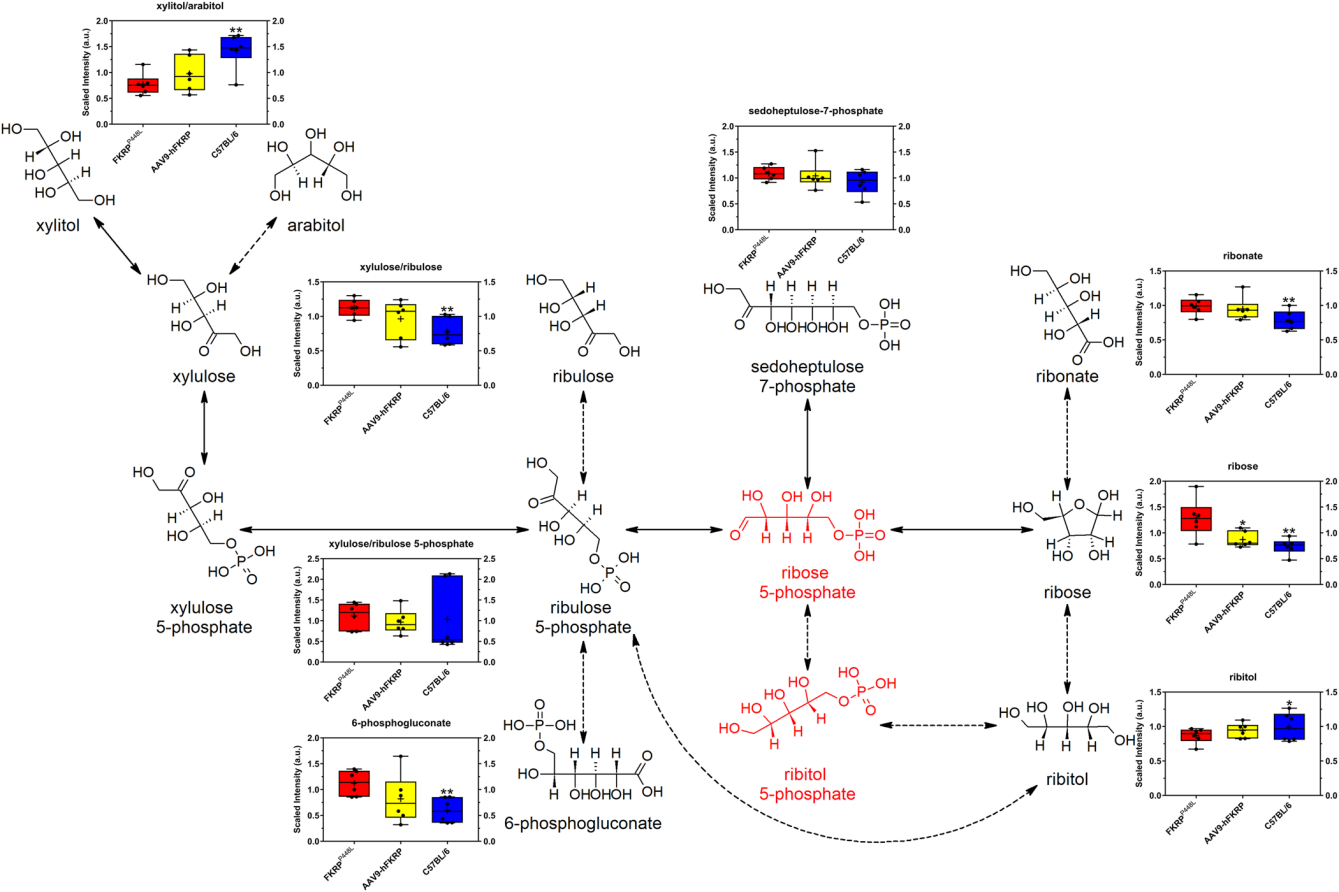
Pentose/pentitol metabolism. Comparison of metabolite abundances in quadriceps muscles acquired from 52-week old untreated FKRP^P448L^ (red), AAV9-hFKRP-treated (yellow), and C57BL/6 (blue) mice. The dashed arrows indicate conversions to sugars and polyols that have not yet been verified in humans. Metabolites in red were either not detected or not in the metabolite library. *p ≤ 0.05, **p ≤ 0.01, ***p ≤ 0.001; p values were determined by two-way ANOVA with repeated measurements.

Noticeable changes in analyte levels associated with the pentose phosphate pathway were observed between untreated FKRP^P448L^ and C57BL/6 skeletal muscle samples, which is expected given the functional activity of FKRP as a ribitol 5-phosphate transferase. Specifically, the levels of ribose (1.74-fold of C57BL/6, p = 1.50 × 10^−3^), ribonate (1.26-fold of C57BL/6, p = 7.60 × 10^−3^), ribulose/xylulose (1.44-fold of C57BL/6, p = 8.10 × 10^−3^), and 6-phosphogluconate (1.88-fold of C57BL/6, p = 5.80 × 10^−3^) were significantly higher in the untreated FKRP^P448L^ muscles, whereas ribitol (0.87-fold of C57BL/6, p = 2.76 × 10^−2^), arabitol/xylitol (0.54-fold of C57BL/6, p = 3.90 × 10^−3^), and arabonate/xylonate (0.58-fold of C57BL/6, p = 9.80 × 10^−3^) were significantly lower (Fig. 3 and Supplementary Dataset). When FKRP^P448L^ mice were administered AAV9-hFKRP, the long-term treatment allowed for limited restoration of the pentose/pentitol metabolism, and only levels of ribose (0.58-fold of untreated FKRP^P448L^, p = 1.68 × 10^−2^) were significantly reduced. These results demonstrate that pentose/pentitol metabolism is altered in skeletal muscles derived from FKRP^P448L^ mice and that FKRP gene therapy can minimally affect these perturbations.

**Figure 4:**
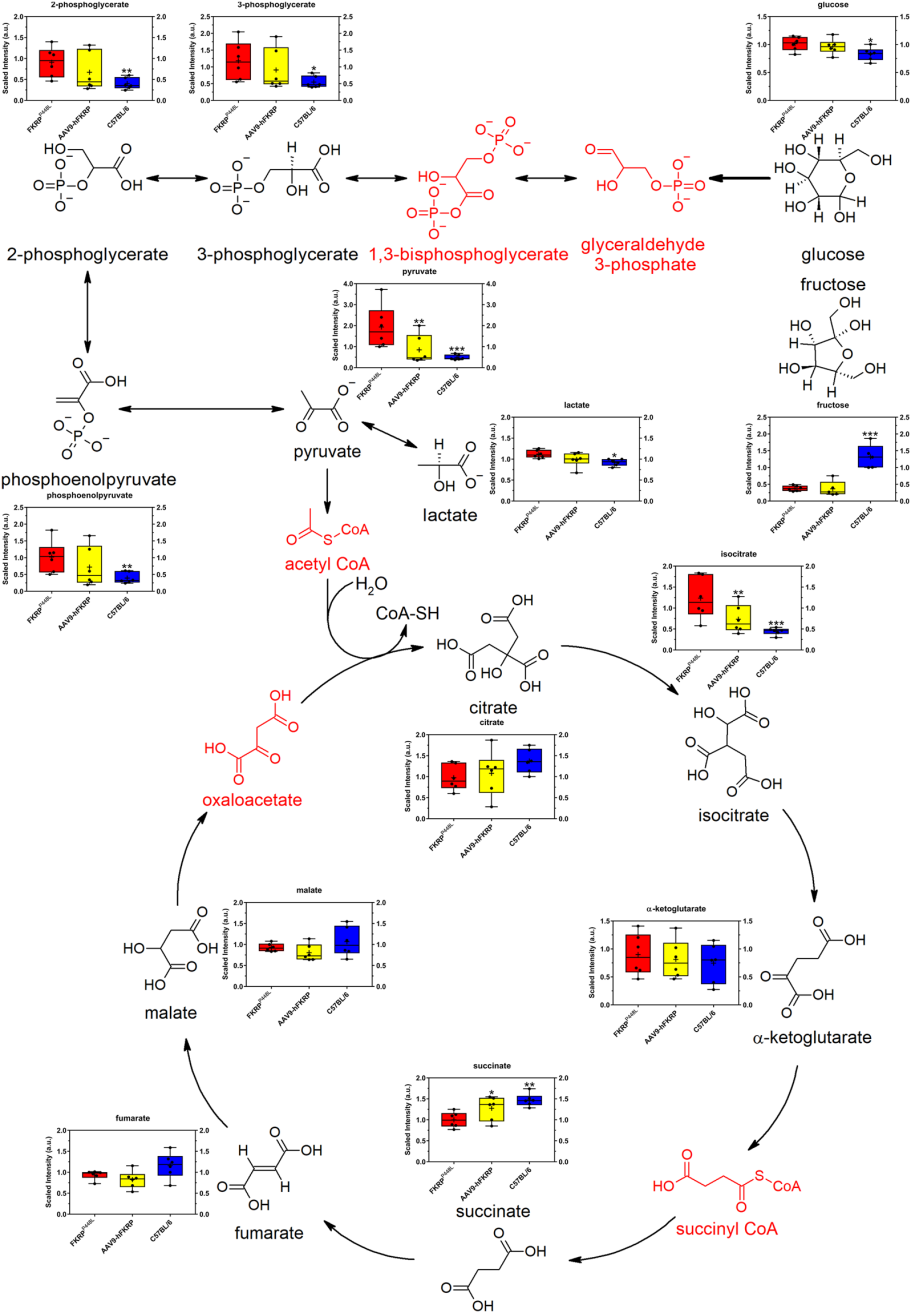
Glycolysis (energy-releasing phase) and TCA cycle. Comparison of metabolite abundances in quadriceps muscles acquired from 52-week old untreated FKRP^P448L^ (red), AAV9-hFKRP-treated (yellow), and C57BL/6 (blue) mice. Metabolites in red were either not detected or not in the metabolite library. *p ≤ 0.05, **p ≤ 0.01, ***p ≤ 0.001; p values were determined by two-way ANOVA with repeated measurements.

In addition to alterations in the levels of pentoses and pentitols, multiple metabolites involved in glycolysis were altered in FKRP^P448L^ mice. More specifically, the second half of glycolysis (energy-releasing phase) appears to be affected (Fig. 4). Elevated levels of glucose (1.22-fold of C57BL/6, p = 3.11 × 10^−2^) and several glycolytic intermediates (e.g., 3-phosphoglycerate (2.15-fold of C57BL/6, p = 1.65 × 10^−2^), 2-phosphoglycerate (2.27-fold of C57BL/6, p = 4.70 × 10^−3^), phosphoenolpyruvate (2.59-fold of C57BL/6, p = 4.40 × 10^−3^), pyruvate (3.97-fold of C57BL/6, p = 2.00 × 10^−4^), and lactate (1.21-fold of C57BL/6, p = 3.66 × 10^−2^)) were detected in skeletal muscles of untreated FKRP^P448L^ mice, but these levels trended back towards those observed in C57BL/6 mice after FKRP gene therapy. These changes, together with significantly reduced levels of fructose in both untreated FKRP^P448L^ (0.19-fold of C57BL/6, p = 4.00 × 10^−4^) and AAV9-hFKRP-treated (0.30-fold of C57BL/6, p = 9.00 × 10^−4^) skeletal muscles may point to increased utilization of the glycolytic pathway because of higher energetic demands required to repair the damaged skeletal muscle (Fig. 4). In turn, the tricarboxylic acid (TCA) cycle is also affected to some degree in the untreated FKRP^P448L^ mice (Fig. 4), with an elevation in pyruvate and isocitrate (2.77-fold of C57BL/6, p = 9.02 × 10^−5^) levels and a decline in succinate (0.68-fold of C57BL/6, p = 1.10 × 10^−3^) levels. All other metabolites involved in the TCA cycle are seemingly unaffected. Despite FKRP gene therapy-induced decreases in isocitrate (0.59-fold of untreated FKRP^P448L^, p = 8.00 × 10^−3^) and increases in succinate (1.28-fold of untreated FKRP^P448L^, p = 2.68 × 10^−2^), the levels of citrate, α-ketoglutarate, fumarate, and malate were largely unaffected (Fig. 4 and Supplementary Dataset). Additionally, alterations in tricarboxylic acids and derivates thereof were observed, with tricarballylate (1,2,3-propanetricarboxylic acid) (0.50-fold of C57BL/6, p = 2.10 × 10^−3^) and 2-methylcitrate/homocitrate (0.63-fold of C57BL/6, p = 1.39 × 10^−2^) levels being significantly lower in untreated FKRP^P448L^ mice, which may cause some additional interference within the TCA cycle.

The metabolic aberrations associated with carbohydrate metabolism collectively demonstrate that these pathways are severely perturbed in FKRP^P448L^ skeletal muscle, and FKRP gene therapy can, to a certain extent, re-establish normal levels of the intermediates involved in each process. These results suggest that there is an increased reliance on glucose as an energy source and possible perturbations in the bioenergetics of the dystrophic muscle, which may be due to increased energy consumption, mitochondrial dysfunction, and/or differences in fatty acid metabolism (discussed in greater detail in the next section).

### Lipid metabolism

**Figure 5:**
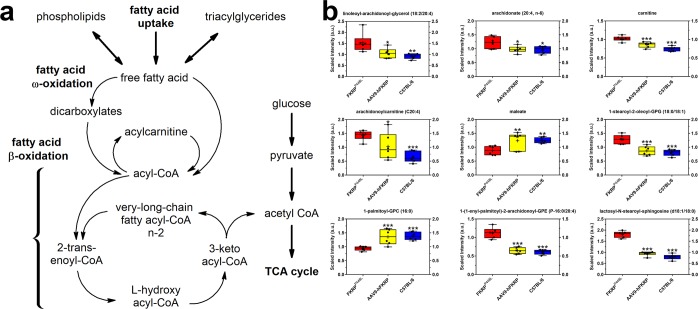
Lipid metabolism. (**a**) Overview of the possible intracellular pathways for fatty acids. (**b**) Comparison of metabolite abundances in quadriceps muscles acquired from 52-week old untreated FKRP^P448L^ (red), AAV9-hFKRP-treated (yellow), and C57BL/6 (blue) mice. *p ≤ 0.05, **p ≤ 0.01, ***p ≤ 0.001; p values were determined by two-way ANOVA with repeated measurements.

Fatty acids are another critical source of energy for mitochondrial oxidation and cellular adenosine triphosphate (ATP) generation, in addition to being precursors for acetyl CoA, phospholipids, and storage lipids. As a result, lipid uptake and subsequent oxidation of fatty acids is a key metabolic pathway that provides energy to support skeletal muscle contractile function and serves an important role in systemic homeostasis (Fig. 5a). Variability in the abundance of multiple diacylglycerols, monounsaturated fatty acids (e.g., palmitoleate [16:1 (n-7)], 10-heptadecenoate [17:1 (n-7)], oleate/vaccenate [18:1]), and polyunsaturated fatty acids (e.g., docosahexaenoate [22:6 (n-3)], arachidonate [20:4 (n-6)], and mead acid [20:3 (n-9)]) were observed in untreated FKRP^P448L^ skeletal muscles (Fig. 5b and Supplementary Dataset), which could suggest an altered lipid metabolism and/or increased hydrolysis of triacylglycerols—too complex for measurement on the DiscoveryHD4™ platform used in this study. Additionally, carnitine (1.36-fold of C57BL/6, p = 2.85 × 10^−6^) and its derivatives, which include multiple acylcarnitines of differing chain-length specificities, exhibited significantly altered abundances in untreated FKRP^P448L^ mice, suggesting inefficient transport across the mitochondrial membrane and/or changes in the regulation of fatty acid β-oxidation (Fig. 5b and Supplementary Dataset). Moreover, levels of dicarboxylates (e.g., glutarate, maleate, pimelate, azelate, and sebacate) generated through ω-oxidation, which is an alternative to β-oxidation, were all lower in untreated FKRP^P448L^ skeletal muscle when compared to C57BL/6 skeletal muscle (Fig. 5b and Supplementary Dataset). Additional comparisons between untreated FKRP^P448L^ and C57BL/6 cohorts reveal that the majority of detectable phosphatidylethanolamines, phosphatidylserines, phosphatidylglycerols, plasmalogens, and sphingomyelins were significantly elevated, whereas the variability in levels of lysophospholipids (LyPs) was based on the moieties associated with the LyP (Fig. 5b and Supplementary Dataset). For example, the LyPs associated with phosphorylcholine and phosphorylethanolamine had significantly lower levels of expression, while LyPs associated with phosphoglycerol moieties were significantly elevated, and LyPs with phosphatidylserine or phosphate moieties were largely unaffected (Supplementary Dataset). Surprisingly, FKRP gene therapy was able to normalize the majority (61%) of these LyPs. Furthermore, the majority of detectable plasmalogens (89%) were significantly elevated in untreated FKRP^P448L^ skeletal muscles when compared to C57BL/6 cohorts, and FKRP gene therapy was able to reverse this trend and normalize almost all of them (Fig. 5b and Supplementary Dataset). Other phospholipid molecules, including multiple phosphatidylcholines and ceramides, were significantly elevated as well as a result of disease progression but responded quite well to FKRP gene therapy (Fig. 5b and Supplementary Dataset). Overall, FKRP gene therapy was able to normalize approximately 46% of the lipid metabolic perturbations, suggesting a potential improvement in transport and/or oxidation of fatty acids.

### Methionine and polyamine metabolism

**Figure 6:**
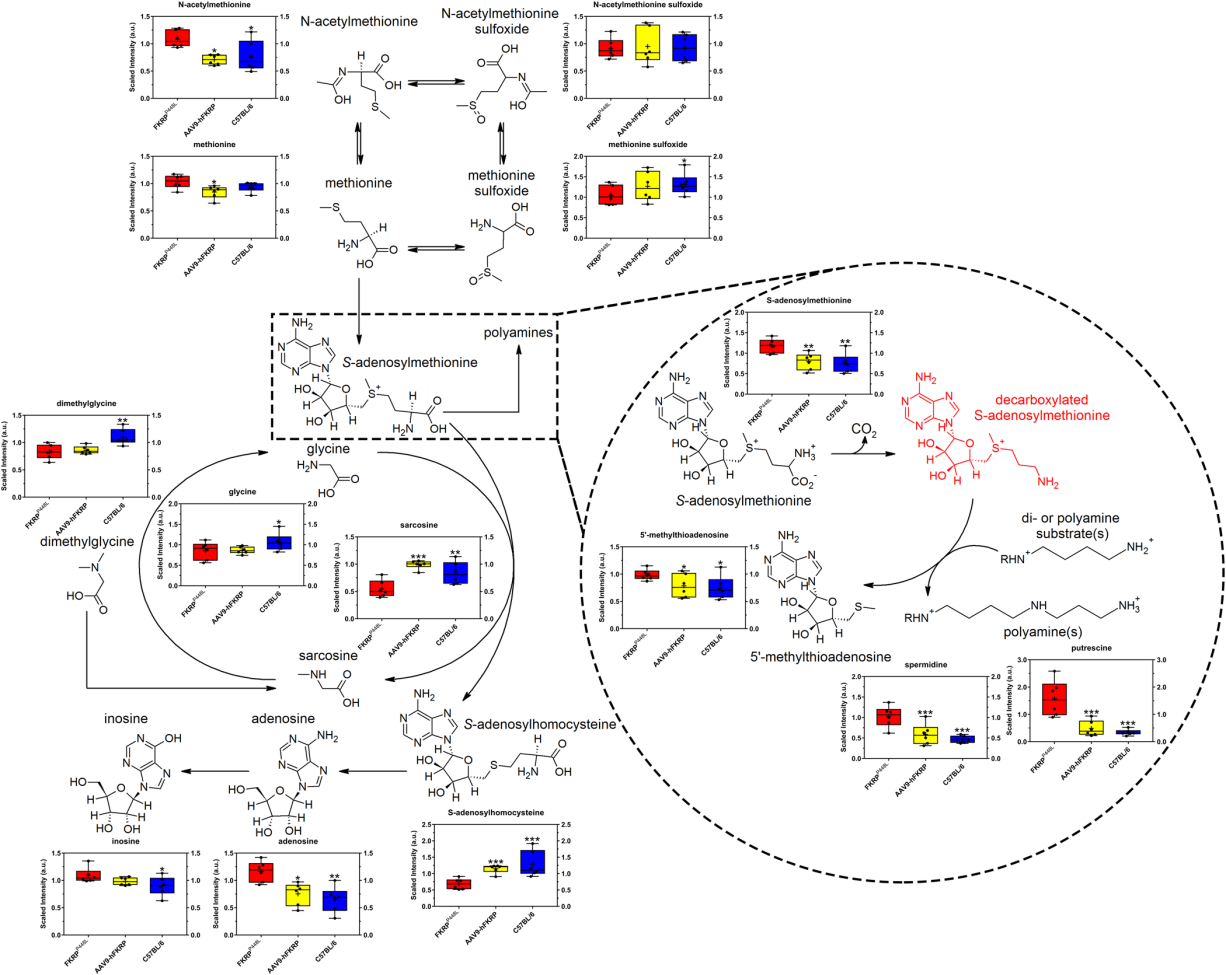
Methionine and polyamine metabolism. Comparison of metabolite abundances in quadriceps muscles acquired from 52-week old untreated FKRP^P448L^ (red), AAV9-hFKRP-treated (yellow), and C57BL/6 (blue) mice. Metabolites in red were either not detected or not in the metabolite library. *p ≤ 0.05, **p ≤ 0.01, ***p ≤ 0.001; p values were determined by two-way ANOVA with repeated measurements.

Finally, significant alterations in metabolites involved in methionine metabolism were also observed, as demonstrated by the accumulation of N-acetylmethionine (1.41-fold of C57BL/6, p = 2.06 × 10^−2^) and *S*-adenosylmethionine (1.55-fold of C57BL/6, p = 2.70 × 10^−3^) and reduced levels of *S*-adenosylhomocysteine (0.53-fold of C57BL/6, p = 6.81 × 10^−6^), sarcosine (0.65-fold of C57BL/6, p = 3.60 × 10^−3^), glycine (0.80-fold of C57BL/6, p = 1.10 × 10^−2^), and dimethylglycine (0.75-fold of C57BL/6, p = 1.60 × 10^−3^) in untreated FKRP^P448L^ skeletal muscles (Fig. 6). Furthermore, indications that polyamine metabolism may be activated in response to disturbances in methionine metabolism is supported by changes in putrescine (4.69-fold of C57BL/6, p = 1.95 × 10^−7^), spermidine (2.20-fold of C57BL/6, p = 8.93 × 10^−5^), and 5’-methylthioadenosine (1.32-fold of C57BL/6, p = 1.58 × 10^−2^) levels, which were observed to be significantly elevated in untreated FKRP^P448L^ samples but then normalized after treatment with FKRP gene therapy (Fig. 6).

## Discussion

Despite knowledge of the primary genetic defects and well-documented histopathology associated with *FKRP*-related dystroglycanopathies, the biomolecular pathogenesis linking the genotype to the wide variation in phenotypes remains poorly understood. And while the variable phenotypic severity has been partly attributed to the differences in mutations within the coding sequence possibly leading to alternation in protein translocation and/or its putative glycosyltransferase activity^3–5^, the possibility of abnormal alternative substrate metabolism cannot be ruled out as a secondary causative factor. In dystrophic skeletal muscle, there is considerable muscle fiber necrosis and attenuated muscle regeneration, a proliferation of connective/adipose tissue, infiltration of immune cells, activation of apoptotic pathways, and altered metabolic capacity, which can have a profound effect on the molecular pathology^27,28^. To the best of our knowledge, this is the first comprehensive metabolomics study of an *FKRP*-related dystroglycanopathy animal model, providing extensive detail about the metabolic alterations that occur in the dystrophic muscle as a result of disease progression and in response to therapeutic intervention with FKRP gene therapy. We have determined that the biomolecular snapshot of the dystrophic skeletal muscle differs substantially from that of normal, unaffected muscle. More importantly, systemic restoration of FKRP protein activity with an AAV9-hFKRP vector was able to correct many of these metabolic impairments.

Not surprisingly, metabolic alterations associated with protein degradation, extracellular matrix remodeling, and/or aging were highlighted by our results. In particular, we observed increased levels of various free N-acetylated amino acids suggesting protein hyperacetylation, which can promote protein degradation or the prevention of translocation^29–31^. Thus, when the level of protein acetylation is imbalanced and protein degradation rates begin to exceed those of protein synthesis, then a loss in muscle mass can occur^31^. Additionally, other studies have shown that dysregulation of N-acetylation results in many severe pathological conditions such as cancers, X-linked genetic disorders, and neurodegenerative diseases^32^. However, the exact biological role and significance of free amino acids and/or hyperacetylated proteins remain complex and enigmatic, and subsequent use of these metabolites as biological markers should be cautioned. Similarly, elevated levels of dipeptides may have some physiological or cell-signaling effects, although most are simply short-lived intermediates on their way to specific amino acid degradation pathways following proteolysis. Other metabolites such as 1-methylhistidine and 3-methylhistidine were significantly dysregulated in *FKRP*-deficient skeletal muscle and are considered potential biomarkers of muscle toxicity^33^. These metabolites constitute an integral part of skeletal muscle contractility and homeostasis, presumably through their role in antioxidant and anti-glycation activities, pH-buffering, or as a calcium regulator^34^. Unfortunately, 1-methylhistidine is not formed in humans, but it does occur in the skeletal muscle of several other species and can serve as a useful biomarker in specific instances. Conversely, 3-methylhistidine is produced in humans, and its presence can reflect the breakdown of contractile elements (e.g., actin and myosin) in various muscle types; however, this process is typically much slower than overall muscle protein degradation^35,36^. Since 3-methylhistidine occurs almost exclusively in muscle actin and myosin and is not reused for protein synthesis or oxidized/metabolized, it has already been proposed as a biomarker of myofibrillar proteolysis, especially in neuromuscular disorders^37,38^. We also observed alterations in some collagen-associated metabolites that could be associated with disease progression and/or accelerated aging. Collagen biosynthesis typically involves an unusually large number of enzyme-catalyzed post-translational modifications, many of which are unique to collagen and a few other proteins with collagen-like amino acid sequences^39,40^. These reactions include the hydroxylation of specific prolyl residues (e.g., trans-4-hydroxyproline) and glycosylation of hydroxylysine residues (e.g., 5-(galactosylhydroxy)-L-lysine). Results highlighted in this study are similar to those in other studies, specifically where levels of trans-4-hydroxyproline in skeletal muscle are reduced with age and where there is an age-associated increase in C-glycosyl tryptophan, which may indirectly reflect increased glycosyltransferase activity against mature proteins^41^. As a result, some of the above-mentioned metabolites may serve as useful biomarkers for evaluating longitudinal changes in muscle biology, including muscle composition, architecture, and/or function.

Another suspected metabolic pathway highlighted by our results was the shift in levels of metabolites associated with carbohydrate metabolism, specifically those involved in pentose/pentitol metabolism and glycolysis. In general, enhancement of the pentose phosphate pathway is important for anabolic processes in the initial stages of skeletal muscle regeneration—a key factor in *FKRP*-deficient dystroglycanopathies^42,43^. However, information regarding pentose/pentitol metabolism in mammalian systems is limited despite their existence in a variety of living organisms. Moreover, the measurement of basal pentose/pentitol concentrations is difficult because endogenous concentrations are typically very low, and distinguishability is complicated by similarities in molecular weight and structure. Nevertheless, the metabolic impairments we observed suggest that polyol pathway flux may yield biomarkers of clinical risk associated with *FKRP*-related dystroglycanopathy. Given that FKRP gene therapy has limited effects at restoring the polyol flux, our results suggest that pentose/pentitol supplementation may assist in the reactivation of the pentose phosphate pathway, which is supported by previously published studies^9,10,44^. In addition to pentose metabolism, the *FKRP*-deficient skeletal muscle also showed evidence of altered glucose metabolism. The inflammatory response that typically accompanies regeneration likely results in accelerated glucose utilization for the production of nucleic acids and lipids, which could explain the accumulation of glycolytic intermediates in the dystrophic skeletal muscle, whereas AAV9-hFKRP-treated mice showed a reduction or correction of these metabolites. We also found a lower baseline concentration of fructose in the skeletal muscle of both *FKRP*-deficient and AAV9-hFKRP-treated mice. One theory supported by evidence in human skeletal muscle is that the mouse skeletal muscle is directly metabolizing fructose, which can result when plasma levels and energy demand are both high^45^. These results indicate shifts in carbohydrate metabolism and energy utilization that can result in profound metabolic impairments that most assuredly contribute to the pathogenic mechanisms of *FKRP*-related dystroglycanopathy, thereby warranting further assessment.

One of the most striking differences highlighted by our results were the metabolic impairments associated with lipid metabolism. Besides carbohydrates, free fatty acids are another source of cellular energy, and lipid oxidation is the prime pathway for supplying these energetic demands. In this study, we see indications that fatty acid oxidation is defective in untreated FKRP^P448L^ mice, which could suggest increased energy demands, mitochondrial defects, or even a shift toward glycolysis^46,47^. Thus, when this system becomes unbalanced, such that mitochondrial oxidation is outpaced by the accumulation of intramyocellular lipids, then elevated levels of intermediary metabolites (e.g., triglyceride, fatty acyl-CoA, diacylglycerols, and ceramides) are expected^48^, which was evident to a certain degree in this study. In addition, long-chain fatty acids must be conjugated to carnitine for efficient transport across the mitochondrial membrane to occur. Yet, levels of carnitine are significantly dysregulated in *FKRP*-deficient skeletal muscle, which suggests an aberration in skeletal muscle mitochondrial function. If this is the case and free long-chain fatty acids are accumulating in the cell, then their use as a fuel source is severely compromised and may be a likely contributor to the presented phenotype. This link between genotype and phenotype, as it relates to long-chain fatty acid β-oxidation, has been previously demonstrated in very-long-chain acyl-CoA dehydrogenase (VLCAD) deficiencies, which present as three clinically heterogeneous phenotypes that are eerily similar to those exhibited by *FKRP*-related dystroglycanopathies^49,50^. Moreover, these metabolic differences are typically reflective of extensive inflammatory cell infiltration in the skeletal muscle in response to excess fatty acids and ectopic lipid deposition. If these autonomous inflammatory responses are allowed to propagate, then chronic inflammation, fibrosis, and accumulation of intramuscular adipose can occur^51^. Consequently, all of these factors create a link between mitochondrial dysfunction, lipotoxicity, and oxidative stress, which, in turn, may impact the metabolic profile of the skeletal muscle. Given the significant alterations observed for energy metabolism, additional assessment of fatty acid metabolism and mitochondrial lipid trafficking in *FKRP*-deficient cells and/or tissues could help shed light on the effects of disease progression and responses to therapeutic intervention as they relate to cellular bioenergetics.

Another pathway that was clearly affected in FKRP^P448L^ mice was methionine and polyamine metabolism. Reduced levels in a few metabolites downstream (e.g., sarcosine, glycine, and *S*-adenosylhomocysteine) in the methionine pathway suggest that resources are being diverted towards the polyamine metabolism pathway. Specifically, our data indicate that increased levels of *S*-adenosylmethionine produce highly elevated levels of polyamines (e.g., putrescine and spermidine) and 5′-methylthioadenosine. Because polyamines are negatively charged polycations with antioxidant properties that can interact with DNA, RNA, or proteins, increased intracellular levels of these metabolites may reflect cell degeneration and regeneration, thereby disrupting homeostatic regulation of energy and glucose metabolism^52^. Additionally, changes in 5′-methylthioadenosine abundance might also reflect differences related to antioxidant function in muscle maintenance and regeneration. Given that AAV9-hFKRP treatment corrected these metabolic impairments, measurement of polyamine-associated metabolites may be useful biomarkers of disease progression and efficacy during gene therapy.

Although not typically classified as a metabolic disorder, there is evidence to suggest that muscular dystrophy-dystroglycanopathies are characterized by perturbed metabolic networks. Some of the metabolic pathways referenced in this study have been identified previously in similar physiological disorders, including anomalies in amino acid, glucose, lipid, and mitochondrial metabolism^47,53^. Using our data set derived from skeletal muscle tissue extracts from *FKRP*-deficient mice treated with AAV9-hFKRP and the respective positive (C57BL/6) and negative (FKRP^P448L^) controls, we have been able to expand the current metabolic footprint of musculoskeletal disorders and identify new potential candidate biomarkers in skeletal muscle associated with FKRP deficiency and age-related muscle pathology. Additionally, we have compiled a list of the top 20 metabolites, which is dominated by lipids but is also highlighted by contributors of amino acid or nucleic acid metabolism (Table 2). More importantly, we find that the majority of these metabolites are fully or partially corrected after FKRP gene therapy. To that end, our study has several limitations that must be acknowledged. First, the non-targeted metabolomics approach was ideal in the assessment of a broad spectrum of cellular macromolecules. However, the ability to conduct quantitative assessment of specific metabolites is hindered. In the future, we plan to narrow our focus to specific pathways using a targeted metabolomics approach to improve our biological understanding in both clinical and preclinical research, especially before intensely pursuing individual metabolites as putative driving factors of skeletal muscle dysfunction in *FKRP*-related dystroglycanopathies. Second, our experimental plan only allowed us to capture a single time-point along the pathologic continuum of *FKRP*-related dystroglycanopathy. While significant differences can be observed at this stage of disease progression and the long-term treatment outcome is one of the most important aspects for clinical application, a more extensive analysis of tissue-specific metabolic alterations at multiple time-points would greatly improve our understanding of the disease etiology. Nevertheless, this exploratory study helps lay the groundwork for future studies related to metabolic deficiencies in *FKRP*-related dystroglycanopathies.

**Table 2:** Top 20 metabolites.

Metabolite	Metabolic Pathway	Sub-Pathway	HMDB^a^	Fold Change	p-value	q-value	Degree of Correction^b^
lactosyl-N-stearoyl-sphingosine (d18:1/18:0)	Lipid	Ceramides	HMDB0011591	2.29	1.12E-08	2.25E-06	Partial
glycerophosphoglycerol	Lipid	Glycerolipid Metabolism	n/a	2.02	2.02E-08	2.25E-06	Partial
1-palmitoyl-GPC (16:0)	Lipid	Lysophospholipid	HMDB0010382	0.68	3.41E-08	2.25E-06	Normalized
1-palmitoyl-GPE (16:0)	Lipid	Lysophospholipid	HMDB0011503	0.52	3.48E-08	2.25E-06	Normalized
5-(galactosylhydroxy)-L-lysine	Amino Acid	Lysine Metabolism	HMDB0000600	2.24	4.44E-08	2.30E-06	Partial
1-palmitoyl-GPS (16:0)	Lipid	Lysophospholipid	n/a	0.24	1.34E-07	4.95E-06	Partial
putrescine	Amino Acid	Polyamine Metabolism	HMDB0001414	4.69	1.95E-07	5.61E-06	Normalized
palmitoyl sphingomyelin (d18:1/16:0)	Lipid	Sphingolipid Metabolism	HMDB0010169	1.47	2.28E-07	5.90E-06	Partial
1-stearoyl-2-oleoyl-GPG (18:0/18:1)	Lipid	Phosphatidylglycerol (PG)	HMDB0010604	1.59	2.84E-07	6.68E-06	Normalized
2-hydroxyadipate	Lipid	Fatty Acid, Dicarboxylate	HMDB0000321	0.50	4.71E-07	9.82E-06	Unchanged
glycerophosphorylcholine (GPC)	Lipid	Phospholipid Metabolism	HMDB0000086	1.61	4.93E-07	9.82E-06	Partial
1-palmitoyl-2-oleoyl-GPG (16:0/18:1)	Lipid	Phosphatidylglycerol (PG)	HMDB0010574	1.56	5.84E-07	1.08E-05	Partial
1-linoleoyl-GPE (18:2)	Lipid	Lysophospholipid	HMDB0011507	0.61	6.30E-07	1.09E-05	Partial
imidazole lactate	Amino Acid	Histidine Metabolism	HMDB0002320	1.58	9.68E-07	1.56E-05	Partial
1-(1-enyl-palmitoyl)-2-arachidonoyl-GPE (P-16:0/20:4)	Lipid	Plasmalogen	HMDB0011352	1.85	1.12E-06	1.70E-05	Normalized
butyrylcarnitine	Lipid	Fatty Acid Metabolism (also BCAA Metabolism)	HMDB0002013	0.33	1.62E-06	2.33E-05	Partial
2’-deoxyuridine	Nucleotide	Pyrimidine Metabolism, Uracil containing	HMDB0000012	0.31	1.83E-06	2.49E-05	Unchanged
carnitine	Lipid	Carnitine Metabolism	HMDB0000062	1.36	2.85E-06	3.51E-05	Partial
orotidine	Nucleotide	Pyrimidine Metabolism, Orotate containing	HMDB0000788	1.67	3.35E-06	3.85E-05	Normalized
C-glycosyltryptophan	Amino Acid	Tryptophan Metabolism	n/a	2.05	3.42E-06	3.85E-05	Normalized

*t*-Test analysis reveals the metabolites with the highest contributions to the metabolic profile ranked by p-value in the comparison of untreated FKRP^P448L^ versus C57BL/6.

^a^Human metabolome database (www.hmdb.ca).

^b^Degree of correction in the metabolite levels after treatment with AAV9-hFKRP.

Currently, the diagnosis of dystroglycanopathy patients is based on the results of a thorough clinical evaluation, which can include laboratory tests for serum enzymes characteristic of the neuromuscular diseases (e.g., creatine kinase (CK), lactic dehydrogenase (LDH), and aldolase), brain and muscle imaging (e.g., magnetic resonance imaging (MRI), computerized tomography (CT), or ultrasound), histological examination of muscle biopsies, and genetic testing. Nevertheless, some of these indicators can have considerable patient-to-patient variability and, at times, may lead to a potential misdiagnosis. Hence, the use of more advanced omics-based strategies should aid in the identification of more specific clinical biomarkers, thereby creating a more patient-specific approach. As this type of technology advances and omics-based tests become more sophisticated, biochemical analysis will continue its prominent role to provide a fundamental basis for future molecular diagnostics as it relates to the physiological context of muscular dystrophy-dystroglycanopathies and a patient-specific hierarchy of clinically actionable pathways for therapeutic interventions.

## Methods

### Study design

This was an open-label, non-randomized study designed to search for possible differences among experimental treatment groups. Technical/biological replicates were performed for validation of formal analysis. All mice were handled according to the Office of Laboratory Animal Welfare guidelines for the humane care and use of experimental animals, and all studies were approved by the Institutional Animal Care and Use Committee (IACUC) of Carolinas Medical Center (Charlotte, NC). Animals were housed in individually ventilated cages (Tecniplast, West Chester, PA) and the photoperiod was a 12-hour light/12-hour dark cycle. Mice were provided *ad libitum* access to food (Teklad Global 18% Protein Rodent Diet, Envigo, Madison, WI) and water.

### Mouse models

FKRP^P448L^ mice were generated by the McColl-Lockwood Laboratory for Muscular Dystrophy Research, as previously described^24,25^. These mice contain a homozygous missense mutation (c.1343 C > T, p.Pro448Leu) in the *FKRP* gene with the floxed neomycin resistant (Neo^r^) cassette removed from the insertion site. FKRP^P448L^ mice become symptomatic at a very young age (approximately 3–4 weeks) and display a mild-to-moderate phenotype throughout the lifespan. C57BL/6 (wild-type) mice were originally obtained from the Jackson Laboratory (Bar Harbor, ME) and used as normal controls where appropriate.

### AAV vector and administration

The AAV9-hFKRP vector was acquired from ViGene Biosciences (Rockville, MD). Further details about these viruses and their packaging and purification can be found on the company website (www.vigenebio.com). Full-length human FKRP cDNA was synthesized for high expression in mouse and subsequently subcloned into a single-stranded AAV9 vector under control of a muscle-specific promoter, followed by a polyadenylation signal from the bovine growth hormone gene. The stock concentration of viral vectors was 2.08 × 10^14^ genome copies per mL and was stored at −80 °C until future use. AAV9-hFKRP diluted in 0.9% sterile saline (minimum volume of 50 µL) was given as a single injection at a dose of 5 × 10^13^ vg/kg into the tail vein of FKRP^P448L^ mice. Mice were injected at 5 weeks of age (n = 6) and sacrificed upon reaching 52 weeks of age. Untreated FKRP^P448L^ and C57BL/6 mice (n = 6 for each cohort) were euthanized at the same age point as the respective AAV9-hFKRP-treated cohorts. All animal cohorts were assessed with an equal number of male & female mice.

### Global metabolomics

Non-targeted global metabolomic profiling of quadriceps muscles derived from AAV9-hFKRP-treated FKRP^P448L^, untreated FKRP^P448L^, and C57BL/6 mice was performed by Metabolon (Durham, NC, USA), according to published methods (more detail in the Supplementary Information)^54^. In brief, neat methanol, containing select isotopically-labeled internal standards, was used to precipitate all the macromolecules (DNA, RNA, and protein) in the biological matrix. The purified supernatant was divided into aliquots corresponding to the various analytical methodologies, then subsequently evaporated and reconstituted with the appropriate analytical injection solvent. Samples were analyzed with four separate methods: two positive mode methods (Pos Early UHPLC-RP/MS/MS and Pos Late UHPLC-RP/MS/MS) and two negative mode methods (Neg UHPLC-RP/MS/MS and Neg UHPLC-HILIC/MS/MS) to ensure broad coverage of biochemicals. The information output from the raw data files was automatically extracted and metabolites of known identity were recognized by comparison to metabolomic library entries of purified standards. This list of metabolites was further condensed to include only those that contained analytical values for each biological replicate, providing a total of 524 metabolites. Data are presented as fold change in three comparison groups: (1) Untreated FKRP^P448L^ versus C57BL/6J, (2) AAV9-hFKRP versus C57BL/6J, and (3) AAV9-hFKRP versus Untreated FKRP^P448L^ (metabolite ratio of >0.9, significant (p ≤ 0.05, q ≤ 0.10) increase; metabolite ratio of <1.10, significant (p ≤ 0.05, q ≤ 0.10) decrease).

### Statistical analysis and pathway diagrams

Statistical analysis of log-transformed metabolomic data was performed using two-way ANOVA to assess the treatment effect and adjust for the sex of animals. For the post-hoc contrasts, p-values and false discovery rate (FDR) were calculated according to a previously proposed method^55^. Resulting q-values were assessed across the entire dataset and significance was defined as p ≤ 0.05 and q ≤ 0.10. Data are presented as box-and-whisker plots with Tukey whiskers that show mean ( + ), minimum, 25% quartile, median, 75% quartile, and maximum. Chemical structures were generated using the IUPAC International Chemical Identifier (InChI) in ACD/ChemSketch (Freeware) 2017.2.1.

## Supplementary information

Supplementary Information

Supplementary Dataset 1

## Data Availability

All data generated/analyzed in this study are included in this article or in the Supplementary Information files and can be provided upon request.
