# Supplementary material for: Metabolomics Analysis of Skeletal Muscles from FKRP-Deficient Mice Indicates Improvement After Gene Replacement Therapy
Source: Sci Rep. 2019 Jul 11;9:10070. doi: 10.1038/s41598-019-46431-1 (PMC6624266; doi:10.1038/s41598-019-46431-1)
Supplement: Supplementary file 1 — Supplementary Information [file 41598_2019_46431_MOESM1_ESM.docx]

**SUPPLEMENTARY INFORMATION**

**Metabolomics Analysis of Skeletal Muscles from FKRP-Deficient Mice Indicates Improvement After Gene Replacement Therapy**

Charles Harvey Vannoy,^1*^ Victoria Leroy,^1^ Katarzyna Broniowska,^2^ and Qi Long Lu^1*^

^1^McColl-Lockwood Laboratory for Muscular Dystrophy Research, Carolinas Medical Center, Atrium Health, Charlotte, NC, 28203, USA

^2^Metabolon, Inc., Morrisville, NC 27560, USA

*Correspondence and requests for materials should be addressed to C.H.V. or Q.L.L.

(e-mail: charles.vannoy@atriumhealth.org; qi.lu@atriumhealth.org)

**MATERIALS AND METHODS**

**Sample Accessioning.** Following receipt by Metabolon (Durham, NC, USA), samples were inventoried and immediately stored at -80 ºC. Each sample received was accessioned into the Metabolon laboratory information management system (LIMS) and was assigned by the LIMS a unique identifier that was associated with the original source identifier only. This identifier was used to track all sample handling, tasks, and results. The samples (and all derived aliquots) were tracked by the LIMS system. All portions of any sample were automatically assigned their own unique identifiers by the LIMS when a new task was created; the relationship of these samples was also tracked. All samples were maintained at -80 ºC until processed.

**Sample Preparation.** Samples were prepared using the automated MicroLab STAR® system from Hamilton Company (Reno, NV). Several recovery standards were added prior to the first step in the extraction process for quality control purposes. To remove protein, dissociate small molecules bound to protein or trapped in the precipitated protein matrix, and to recover chemically diverse metabolites, proteins were precipitated with methanol under vigorous shaking for 2 min using a 2000 Geno/Grinder (Glen Mills, Clifton, NJ) followed by centrifugation. The resulting extract was divided into five fractions: two for analysis by two individual reverse phase ultra-high performance liquid chromatography (RP/UHPLC)-MS/MS with electrospray ionization (ESI) in positive ion mode, one for analysis by RP/UHPLC-MS/MS with ESI in negative ion mode, one for analysis by hydrophilic interaction liquid chromatography (HILIC)/UHPLC-MS/MS with ESI in negative ion mode, and one sample was reserved for backup. Samples were placed briefly on a TurboVap® (Zymark, Hopkinton, MA) to remove the organic solvent. The sample extracts were stored overnight under nitrogen before preparation for analysis.

**Quality Assurance/Quality Control (QA/QC).** Several types of controls were analyzed in concert with the experimental samples: a pooled matrix sample generated by taking a small volume of each experimental sample (or alternatively, use of a pool of well-characterized human plasma) served as a technical replicate throughout the data set; extracted water samples served as process blanks; and a cocktail of QC standards that were carefully chosen not to interfere with the measurement of endogenous compounds were spiked into every analyzed sample, allowed instrument performance monitoring and aided chromatographic alignment. Instrument variability was determined by calculating the median relative standard deviation (RSD) for the standards that were added to each sample prior to injection into the mass spectrometers. Overall process variability was determined by calculating the median RSD for all endogenous metabolites (i.e., non-instrument standards) present in 100% of the pooled matrix samples. Experimental samples were randomized across the platform run with QC samples spaced evenly among the injections.

**Ultra-High Performance Liquid Chromatography Tandem Mass Spectrometry (UHPLC-MS/MS).** All methods utilized an ACQUITY UHPLC (Waters, Milford, MA) and a Q Exactive™ high resolution/accurate mass spectrometer interfaced with a heated electrospray ionization (HESI-II) source and Orbitrap mass analyzer (Thermo Fisher Scientific, Waltham, MA) operated at 35,000 mass resolution. The sample extract was dried then reconstituted in solvents compatible to each of the four methods. Each reconstitution solvent contained a series of standards at fixed concentrations to ensure injection and chromatographic consistency. One aliquot was analyzed using acidic positive ion conditions, chromatographically optimized for more hydrophilic compounds. In this method, the extract was gradient eluted from an ACQUITY UPLC BEH C18 Column, 130Å, 1.7 µm, 2.1 mm X 100 mm (Waters) using water and methanol, containing 0.05% perfluoropentanoic acid (PFPA) and 0.1% formic acid (FA). Another aliquot was also analyzed using acidic positive ion conditions, however it was chromatographically optimized for more hydrophobic compounds. In this method, the extract was gradient eluted from the aforementioned C18 column using methanol, acetonitrile, water, 0.05% PFPA, and 0.01% FA and was operated at an overall higher organic content. Another aliquot was analyzed using basic negative ion optimized conditions using a separate, dedicated C18 column. The basic extracts were gradient eluted from the column using methanol and water, however with 6.5 mM ammonium bicarbonate, pH 8.0. The fourth aliquot was analyzed via negative ionization following elution from a ACQUITY UPLC BEH Amide Column, 130Å, 1.7 µm, 2.1 mm X 150 mm (Waters) using a gradient consisting of water and acetonitrile with 10 mM ammonium formate, pH 10.8. The MS analysis alternated between MS and data-dependent MS^n^ scans using dynamic exclusion. The scan range varied slighted between methods but covered 70-1000 m/z. Raw data files are archived and extracted as described below.

**Bioinformatics.** The informatics system consisted of four major components, the LIMS, the data extraction and peak-identification software, data processing tools for quality control and compound identification, and a collection of information interpretation and visualization tools for use by data analysts. The hardware and software foundations for these informatics components were the LAN backbone, and a database server running Oracle 10.2.0.1 Enterprise Edition.

**LIMS.** The purpose of the Metabolon LIMS was to enable fully auditable laboratory automation through a secure, easy to use, and highly specialized system. The scope of the Metabolon LIMS encompasses sample accessioning, sample preparation and instrumental analysis and reporting and advanced data analysis. All of the subsequent software systems are grounded in the LIMS data structures. It has been modified to leverage and interface with the in-house information extraction and data visualization systems, as well as third party instrumentation and data analysis software.

**Data Extraction, Compound Identification, and Curation.** Raw data was extracted, peak-identified, and QC processed using Metabolon’s hardware and software. These systems are built on a web-service platform utilizing Microsoft’s .NET technologies, which run on high-performance application servers and fiber-channel storage arrays in clusters to provide active failover and load-balancing. Compounds were identified by comparison to library entries of purified standards or recurrent unknown entities. Metabolon maintains a library based on authenticated standards that contains the retention time/index (RI), mass to charge ratio (*m/z)*, and chromatographic data (including MS/MS spectral data) on all molecules present in the library. Furthermore, biochemical identifications are based on three criteria: retention index within a narrow RI window of the proposed identification, accurate mass match to the library +/- 10 ppm, and the MS/MS forward and reverse scores between the experimental data and authentic standards. The MS/MS scores are based on a comparison of the ions present in the experimental spectrum to the ions present in the library spectrum. While there may be similarities between these molecules based on one of these factors, the use of all three data points can be utilized to distinguish and differentiate metabolites. More than 3300 commercially available purified standard compounds have been acquired and registered into LIMS for analysis on all platforms for determination of their analytical characteristics. Additional mass spectral entries have been created for structurally unnamed metabolites, which have been identified by virtue of their recurrent nature (both chromatographic and mass spectral). These compounds have the potential to be identified by future acquisition of a matching purified standard or by classical structural analysis. A variety of curation procedures were carried out to ensure that a high-quality data set was made available for statistical analysis and data interpretation. The QC and curation processes were designed to ensure accurate and consistent identification of true chemical entities, and to remove those representing system artifacts, mis-assignments, and background noise. Metabolon data analysts use proprietary visualization and interpretation software to confirm the consistency of peak identification among the various samples. Library matches for each compound were checked for each sample and corrected if necessary.

**Metabolite Quantification and Data Normalization.** Peaks were quantified using area-under-the-curve. For studies spanning multiple days, a data normalization step was performed to correct variation resulting from instrument inter-day tuning differences. Essentially, each compound was corrected in run-day blocks by registering the medians equal to one (1.00) and normalizing each data point proportionately (termed the “block correction”). For studies that did not require more than one day of analysis, no normalization is necessary, other than for purposes of data visualization. In certain instances, biochemical data may have been normalized to an additional factor (e.g., total protein as determined by Bradford assay, osmolality, etc.) to account for differences in metabolite levels due to differences in the amount of material present in each sample.

**
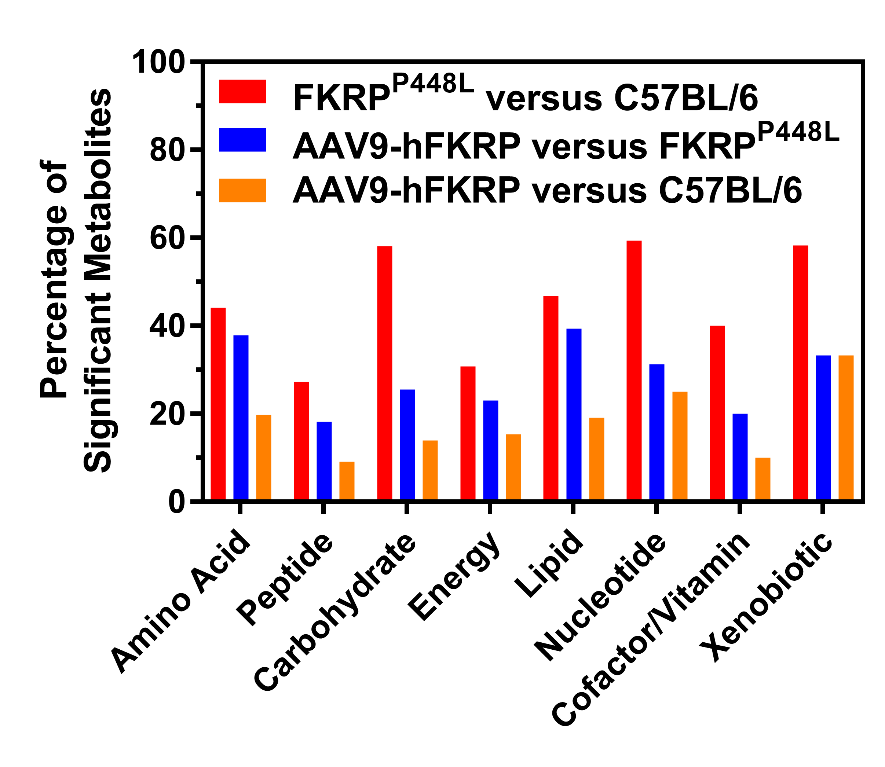
**

**Figure S1. Distribution of metabolites in skeletal muscle.** Comparison analysis detailing the percentage of significantly altered metabolites involved in each respective defined metabolic pathway. Comparison groups: Untreated FKRP^P448L^ versus C57BL/6 (red), AAV9-hFKRP versus Untreated FKRP^P448L^ (blue), and AAV9-hFKRP versus C57BL/6 (orange).

**Table S1. Random Forest confusion matrix.** Random Forest classification using metabolomic profiles derived from the quadriceps muscles (n = 6) in C57BL/6, AAV9-hFKRP-treated, and untreated FKRP^P448L^ mice.

|  | **C57BL/6** | **FKRP^P448L^** | **AAV9-hFKRP** | **Class Error** |
| --- | --- | --- | --- | --- |
| **C57BL/6** | 6 | 0 | 0 | 0 |
| **FKRP^P448L^** | 0 | 6 | 0 | 0 |
| **AAV9-hFKRP** | 0 | 0 | 6 | 0 |
| **Predictive Accuracy ≈ 100%** | | | | |
